# Risk factors for neck hematoma requiring surgical re-intervention after thyroidectomy: a systematic review and meta-analysis

**DOI:** 10.1186/s12893-019-0559-8

**Published:** 2019-07-24

**Authors:** Chunlei Fan, Xin Zhou, Guoqiang Su, Yanming Zhou, Jingjun Su, Mingxu Luo, Hui Li

**Affiliations:** 1 Department of Gastrointestinal Surgery III, Xiamen Cancer Hospital, First Affiliated Hospital of Xiamen University, 55 Zhenhai Road, Xiamen, 361003 China; 2 Department of Hepatobiliary Surgery, First Affiliated Hospital of Xiamen University, Xiamen, China; 3 Department of Ultrasound, First Affiliated Hospital of Xiamen University, 55 Zhenhai Road, Xiamen, 361003 China; 4 Department of Hepatic Surgery and Liver transplantation Center, Third Affiliated Hospital of Sun Yat-sen University, Guangzhou, China

**Keywords:** Risk factor, Hematoma, Bleeding, Thyroidectomy, Thyroid surgery

## Abstract

**Background:**

In this systematic review and meta-analysis, we aimed to determine the risk factors associated with neck hematoma requiring surgical re-intervention after thyroidectomy.

**Methods:**

We systematically searched all articles available in the literature published in PubMed and CNKI databases through May 30, 2017. The quality of these articles was assessed using the Newcastle-Ottawa Quality Assessment Scale, and data were extracted for classification and analysis by focusing on articles related with neck hematoma requiring surgical re-intervention after thyroidectomy. Our meta-analysis was performed according to the Preferred Reporting Items for Systematic Review and Meta-Analyses guidelines.

**Results:**

Of the 1028 screened articles, 26 met the inclusion criteria and were finally analyzed. The factors associated with a high risk of neck hematoma requiring surgical re-intervention after thyroidectomy included male gender (odds ratio [OR]: 1.86, 95% confidence interval [CI]: 1.60–2.17, *P* < 0.00001), age (MD: 4.92, 95% CI: 4.28–5.56, *P* < 0.00001), Graves disease (OR: 1.81, 95% CI: 1.60–2.05, *P* < 0.00001), hypertension (OR: 2.27, 95% CI: 1.43–3.60, *P* = 0.0005), antithrombotic drug use (OR: 1.92, 95% CI: 1.51–2.44, *P* < 0.00001), thyroid procedure in low-volume hospitals (OR: 1.32, 95% CI: 1.12–1.57, *P* = 0.001), prior thyroid surgery (OR: 1.93, 95% CI: 1.11–3.37, *P* = 0.02), bilateral thyroidectomy (OR: 1.19, 95% CI: 1.09–1.30, *P* < 0.0001), and neck dissection (OR: 1.55, 95% CI: 1.23–1.94, *P* = 0.0002). Smoking status (OR: 1.19, 95% CI: 0.99–1.42, *P* = 0.06), malignant tumors (OR: 1.00, 95% CI: 0.83–1.20, *P* = 0.97), and drainage used (OR: 2.02, 95% CI: 0.69–5.89, *P* = 0.20) were not significantly associated with postoperative neck hematoma.

**Conclusion:** We identified certain risk factors for neck hematoma requiring surgical re-intervention after thyroidectomy, including male gender, age, Graves disease, hypertension, antithrombotic agent use, history of thyroid procedures in low-volume hospitals, previous thyroid surgery, bilateral thyroidectomy, and neck dissection. Appropriate intervention measures based on these risk factors may reduce the incidence of postoperative hematoma and yield greater benefits for the patients.

## Background

Thyroidectomy is often required for the treatment of malignant thyroid tumors and some benign thyroid diseases [1]. Although thyroid surgery is a relatively safe procedure, it may be associated with some clinically concerning postoperative complications, including postoperative cervical hematoma, incision infection, hypocalcemia, and in some cases vocal cord paralysis. Of these, the development of postoperative cervical hematoma, although rare (incidence: 0.43–6.54%) [2–27], may lead to symptoms of compression causing airway obstruction, respiratory distress, or even death due to suffocation.

In recent years, due to the development of new instruments, such as bipolar scalpels, ultrasonic shears, and energy platforms, thyroid surgery has become more precise and prevalent on an outpatient basis as a result of short hospital stays and low costs [28]. However, postoperative cervical hematoma remains a potential life-threatening complication, and is the main reason why patients are required to stay in the hospital overnight for monitoring after thyroid surgery. To ensure more widespread application of outpatient thyroidectomy and avoid the potential risk of postoperative cervical hematoma, it is vital to determine risk factors that could help clinical surgeons screen patients suitable for outpatient thyroidectomy, and thus minimize the risk of postoperative hematoma [29].

In the present study, we aimed to identify risk factors associated with neck hematoma requiring surgical re-intervention after thyroidectomy, including the impact of hypertension, hospital volume, and smoking status.

## Methods

Our meta-analysis was performed according to the Preferred Reporting Items for Systematic Review and Meta-Analyses (PRISMA) guidelines [30].

### Study design

This systematic review and meta-analysis was conducted by reviewing relevant retrospective studies and collecting suitable data.

### Search strategies and information sources

We searched all articles published through May 30, 2017, in the PubMed and CNKI databases by using the following key words: “thyroidectomy and hematoma”, “thyroidectomy and hemorrhage”, or “thyroidectomy and postoperative bleeding”. Two authors conducted the search independently, and any disagreement was resolved by discussion with a third individual.

### Study selection

Studies were included in our meta-analysis using the following criteria: a) retrospective studies; b) articles published in the English or Chinese language; c) studies providing data on the clinical characteristics of the patients; d) studies including patients who underwent open thyroidectomy on either an inpatient or outpatient basis; and e) studies including patients with postoperative hematoma requiring surgical re-intervention.

The exclusion criteria were: a) studies with incomplete data or without data that met our inclusion criteria; b) commentaries, letters, and animal studies; and c) studies including patients receiving minimally invasive thyroidectomy or simple parathyroidectomy.

### Data extraction

After identifying the included articles, 2 reviewers were responsible for extracting the data, including the first author, publication time, design of study, number of patients, and incidence. Patient variables included age, sex, procedure type (unilateral, bilateral, with or without neck dissection), pathology, personal characteristics (Graves disease, hypertension, antithrombotic agent use, smoking status, and previous thyroid surgery), and the use of drainage. A re-extraction was performed by the 2 reviewers together via discussion if debatable data were present in any of the studies.

### Quality assessment

Using the Newcastle Ottawa Scale (NOS) ranging from 0 to 9 [31], all the included studies were assessed for the risk of reporting bias by the 2 reviewers. A study with a score of ≥5 was considered as a study of high quality with a low bias risk; otherwise, it was excluded [32–34]. Any disagreement on the final outcome of assessment was resolved through discussion.

### Statistical analysis

The odds ratio (OR) or mean difference (MD) with a 95% confidence interval (CI) was chosen as the parameter for this study, and statistical significance was presented as a polled *p* value< 0.05. The heterogeneity was measured by the Q test and I^2^ statistics. When the heterogeneity test indicated no significant difference (*p* > 0.1 and I^2^ < 50%), a fixed-effect model was used; otherwise, a random-effects model was used, and the possibility for publication and selective reporting bias was presented using a Begg’s funnel plot. All statistical analyses of the data were achieved by using RevMan 5.3 software.

## Results

### Study selection

**Fig. 1:**
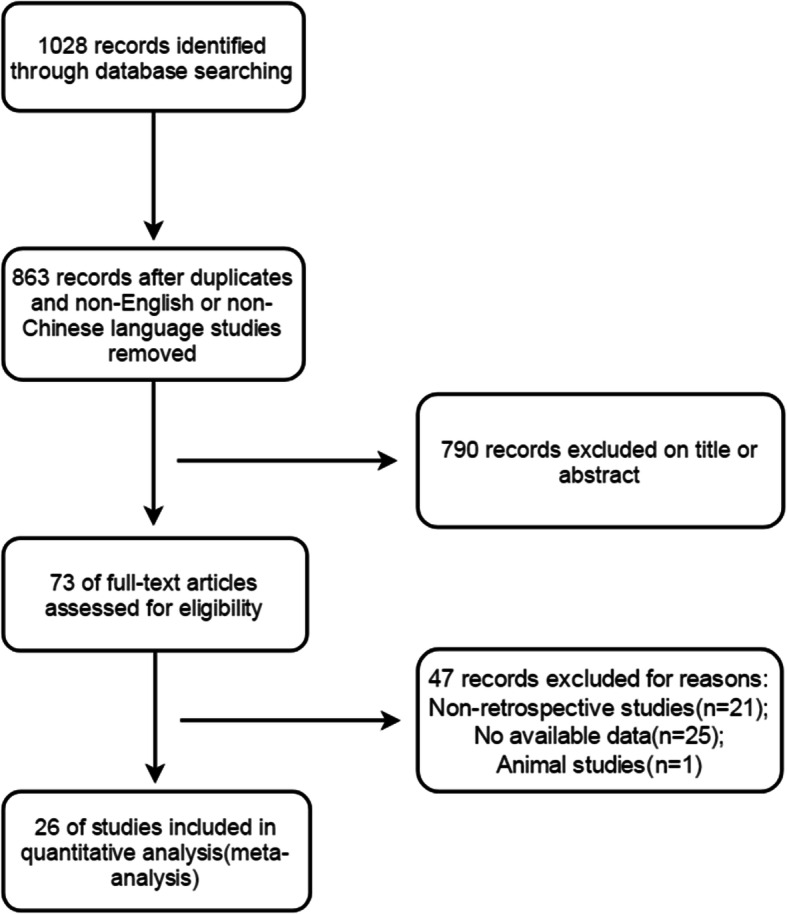
Study flow diagram of the meta-analysis

Through a systematic retrieval and manual search of the databases, 1028 articles were screened. The exclusion criteria included duplicated studies, studies with insufficient data, or studies with data that did not meet the inclusion criteria. Twenty-six studies were deemed eligible for inclusion in the final analysis. The selection process flow chart for the included studies is shown in Fig. 1.

### Study characteristics

The total number of patients included was 452,799, and post-thyroidectomy hematoma requiring surgical intervention was noted in 6663 patients. The basic characteristics of the studies included in our meta-analysis are shown in Table 1.

**Table 1:** The basic characteristics of the included studies

Author	Year	Type of study	Country/Region	NOS score	Age(Mean)	Sex(M/F)	No.Graves	No.Cancer	No.Others	Procedure	Hematoma/Total	Rate(%)
Liu,J.	2016	Retrospective	China	7	NA	1401/3755	0	4167	989	TT,HT	44/5156	0.85
Suzuki,S.	2016	Retrospective	Japan	7	NA	1967/40000	3383	29715	18869	TT,HT	920/51967	1.77
Narayanan,S.	2016	Retrospective	USA	7	51.2	271/1176	NA	441	NA	TT,HT	10/1447	0.69
Oltmann,S.C.	2016	Retrospective	USA	7	49	527/1868	NA	NA	NA	TT,HT	20/2395	0.84
Perera,M.	2016	Retrospective	Australia	7	51.6	41/164	NA	38	NA	TT,HT	9/205	4.39
Sorensen,K.R.	2015	Retrospective	Denmark	7	53.2	378/1134	NA	309	NA	TT,HT	42/1512	2.77
Dehal,A.	2015	Retrospective	USA	7	NA	32033/145124	4342	37038	105954	TT,HT	2210/147334	1.49
Hardman,J.C.	2015	Retrospective	UK	6	46.3	341/1316	NA	NA	NA	TT,HT	32/1657	1.93
Chen,E.	2014	Retrospective	China	7	46.1	872/3577	296	2027	2176	TT,HT	88/4499	1.96
Dixon,J.L.	2014	Retrospective	USA	7	NA	NA	NA	NA	NA	TT,HT	18/4140	0.43
Weiss,A.	2014	Retrospective	USA	7	52	31186/118826	NA	46298	NA	TT,HT	1870/150012	1.25
Vassiliou,I.	2013	Retrospective	Greece	7	54	63/153	2	100	214	TT	3/216	1.39
Kandil,E.	2013	Retrospective	USA	7	NA	NA	1197	9199	11229	TT	366/21625	1.69
Calo,P.G.	2012	Retrospective	Italy	7	NA	8/98	NA	10	NA	TT,HT	3/106	2.83
Promberger,R.	2012	Retrospective	Austria	7	NA	5727/24415	1052	2460	36630	TT,HT	519/30142	1.72
Agarwal,A.	2012	Retrospective	India	7	42.7	244/569	NA	266	NA	TT	6/813	0.74
Lang,B.H.	2012	Retrospective	Hongkong	7	48	569/2517	415	554	2117	TT,HT	22/3086	0.71
Calo,P,G.	2010	Retrospective	Italy	7	56	465/2094	259	799	1501	TT,HT	32/2559	1.25
Godballe,C.	2009	Retrospective	Denmark	7	50	1147/4343	NA	774	NA	TT,HT	230/5422	4.24
Shih,M.L.	2008	Retrospective	USA	7	45.4	82/392	NA	133	NA	TT,HT	4/474	0.84
Leyre,P.	2008	Retrospective	France	7	54	1112/5718	406	NA	NA	TT,HT	70/6830	1.02
Bergenfelz,A.	2008	Retrospective	Sweden	7	NA	659/3001	659	450	2551	TT,HT	76/3660	2.07
Lefevre,J.H.	2007	Retrospective	France	7	51	108/577	NA	92	NA	TT,HT	6/685	0.88
Chiang,F.Y.	2006	Retrospective	Taiwan	6	NA	NA	48	NA	NA	TT	7/107	6.54
Ozlem,N.	2006	Retrospective	Turkey	7	38	180/886	NA	NA	NA	TT,HT	5/1066	0.47
Gaujoux,S.	2006	Retrospective	France	7	49	894/4246	714	NA	NA	TT,HT	51/5140	0.99

*NOS* Newcastle Ottawa Scale, *No*. Number, *M* Male, *F* Female, *NA* Data not available, *TT* Total thyroidectomy; *HT* Hemi−/subtotal/partial thyroidectomy

### Results of individual analysis

#### Gender

**Fig. 2:**
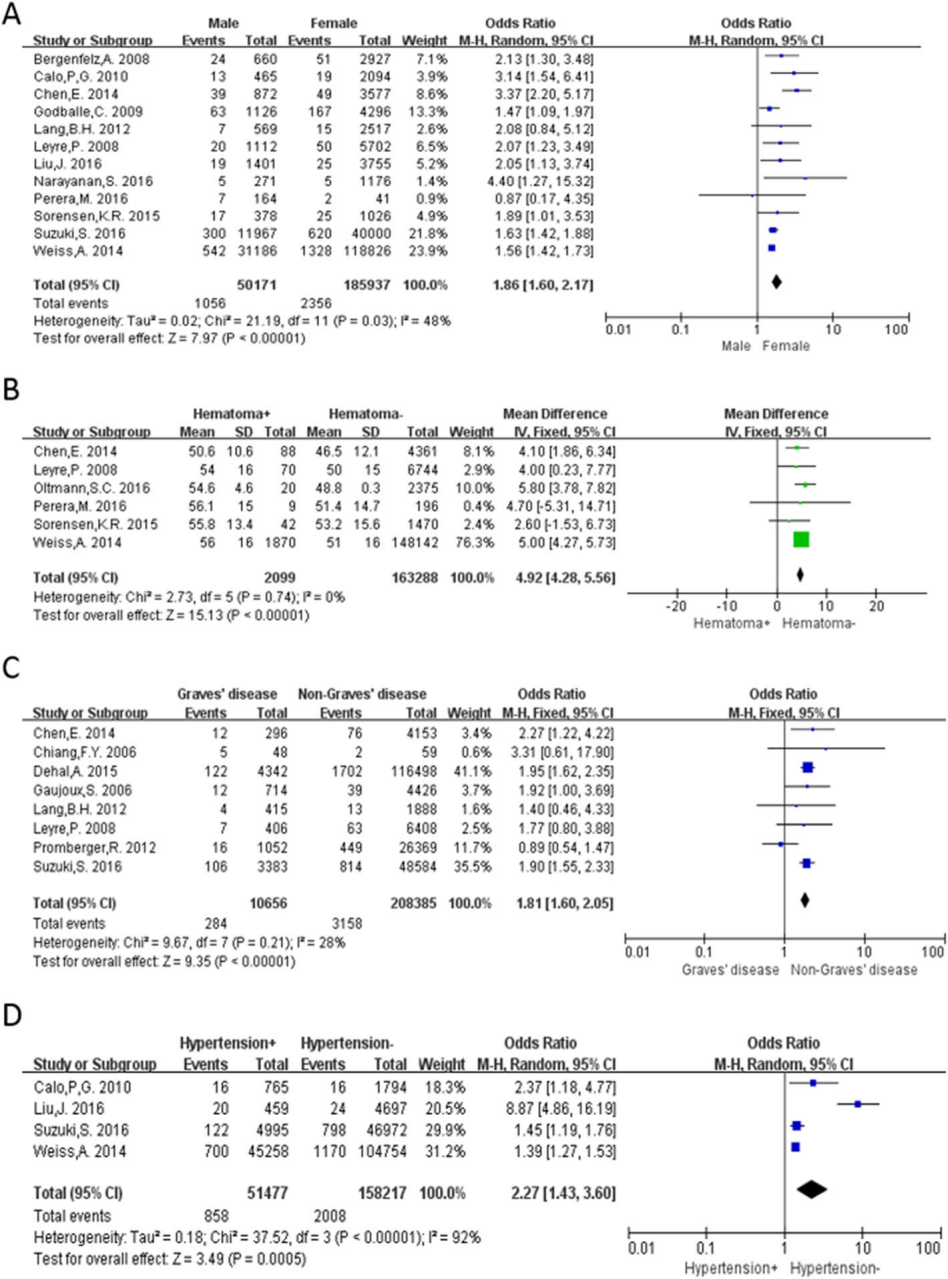
Meta-analysis results of post-thyroidectomy hematoma between the 2 groups. **a** Gender; **b** Age; **c** Graves disease; **d** Hypertension

Twelve studies [2, 3, 5–7, 10, 12, 16, 19, 20, 22, 23] were included for the analysis of hematoma occurrence between males and females. The overall rate of post-thyroidectomy hematoma requiring surgical intervention was 2.10% in males and 1.27% in females. Pooled ORs, using a random-effects model (*P* = 0.03, I^2^ = 48%), showed that the occurrence of postoperative hematoma in males was significantly greater than in females (OR: 1.86, 95% CI: 1.60–2.17, *P* < 0.00001; Fig. 2a).

#### Age

We used a fixed-effects model to analyze the impact of age on the occurrence of postoperative hematoma (*P* = 0.74, I^2^ = 0%). A total of 5 studies [3, 4, 7, 10, 12, 22] were included, which exhibited a significant difference between the hematoma and non-hematoma groups (MD: 4.92, 95% CI: 4.28–5.56, *P* < 0.00001; Fig. 2b). The I^2^ demonstrated that there was no significant heterogeneity between the studies included in the analysis.

#### Graves disease

A fixed-effects model was used for data analysis (*P* = 0.21, I^2^ = 28%), and 8 studies [2, 9, 12, 15, 16, 22, 26, 27] were included in the analysis. There was a significantly higher incidence of post-thyroidectomy hematoma in patients with Graves disease (OR: 1.81, 95%CI: 1.60–2.05, *P* < 0.00001; Fig. 2c).

#### Hypertension

We used a random-effects model for analysis due to the heterogeneity of the data (*P* < 0.00001, I^2^ = 92%). Four studies [2, 6, 10, 19] were included in the analysis. The incidence of postoperative hematoma was significantly greater in the patients with hypertension (OR: 2.27, 95% CI: 1.44–3.68, *P* = 0.0005; Fig. 2d).

#### Antithrombotic drug use

**Fig. 3:**
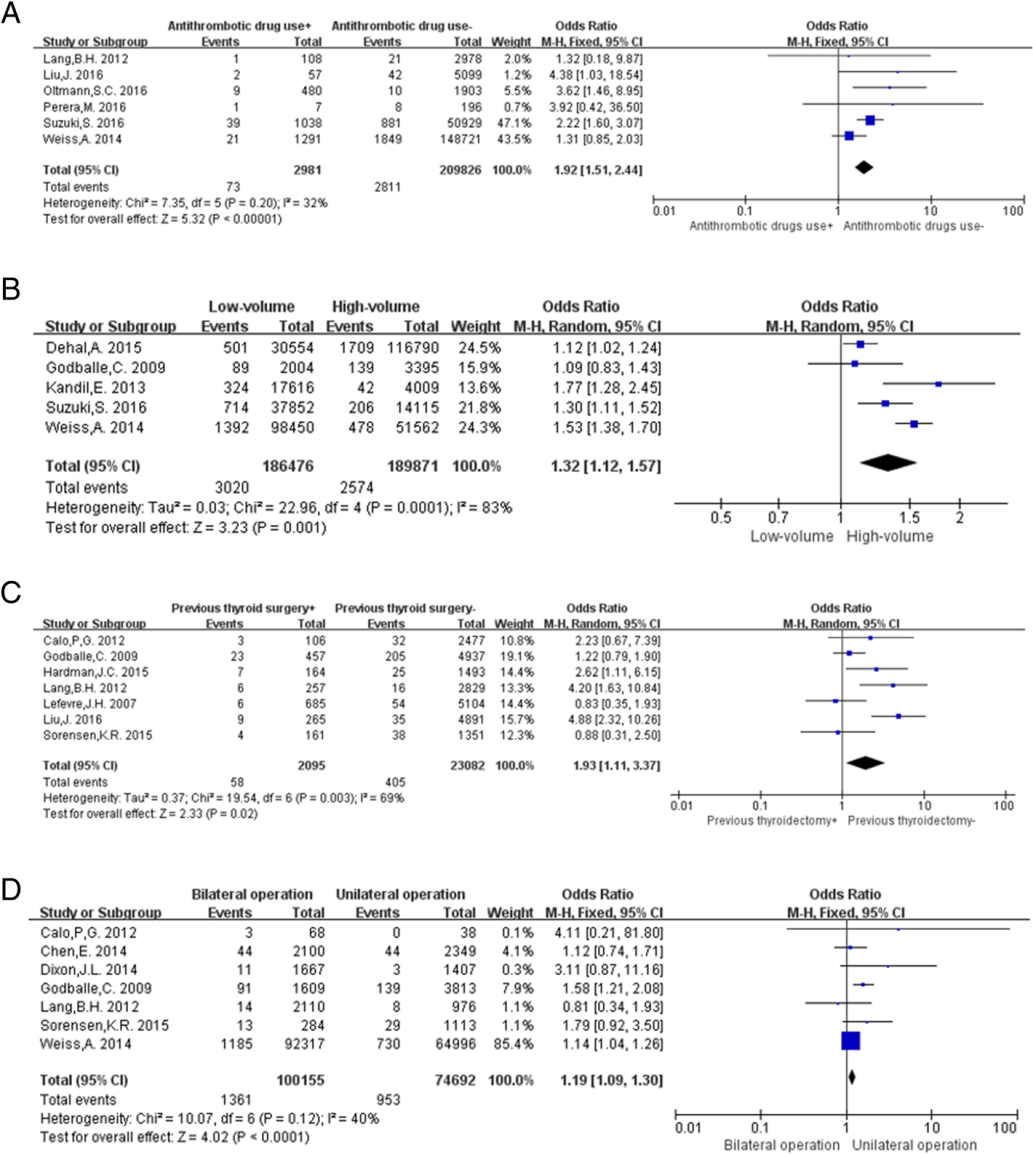
Meta-analysis results of post-thyroidectomy hematoma between the 2 groups. **a** Antithrombotic drug use; **b** Hospital volume; **c** Previous thyroid surgery; **d** Extent of operation

Data concerning the use of antithrombotic drugs were analyzed using a fixed-effects model (*P* = 0.20, I^2^ = 32%). All patients undergoing antithrombotic therapy before the operation were included in this meta-analysis, regardless of the medication route and dosage. The results showed that the occurrence of post-thyroidectomy hematoma in patients using antithrombotic drugs was significantly higher than that in patients who did not use antithrombotic drugs (OR: 1.92, 95% CI: 1.51–2.44, *P* < 0.00001; Fig. 3a).

#### Hospital volume

Hospital volumes in terms of thyroidectomies performed were classified as low (< 75 per year) and high (≥75 per year). Data analysis using the random-effects model (*P* = 0.0001, I^2^ = 83%) showed that thyroidectomies performed in low-volume hospitals had a higher propensity to result in postoperative hematoma, as compared to those performed in high-volume hospitals (OR: 1.32, 95% CI: 1.12–1.57, *P* = 0.001; Fig. 3b).

#### Previous thyroid operation

A random-effects model was used to conduct the data analysis (*P* = 0.003, I^2^ = 69%). Seven studies [6–8, 16, 17, 20, 24] were included in this analysis. The occurrence of post-thyroidectomy hematoma was higher in patients with a history of thyroidectomy than in patients without (OR: 1.93, 95% CI: 1.11–3.37, *P* = 0.02; Fig. 3c).

#### Surgical extent

Based on the extent of surgery, patients were divided into 2 groups: the bilateral surgery group and unilateral surgery group. Data analysis using a fixed-effects model (*P* = 0.12, I^2^ = 40%) showed that postoperative hematoma was more likely to occur in patients receiving bilateral thyroidectomy than in patients receiving unilateral surgery (OR: 1.19, 95% CI: 1.09–1.30, *P* < 0.0001; Fig. 3d).

#### Neck dissection

**Fig. 4:**
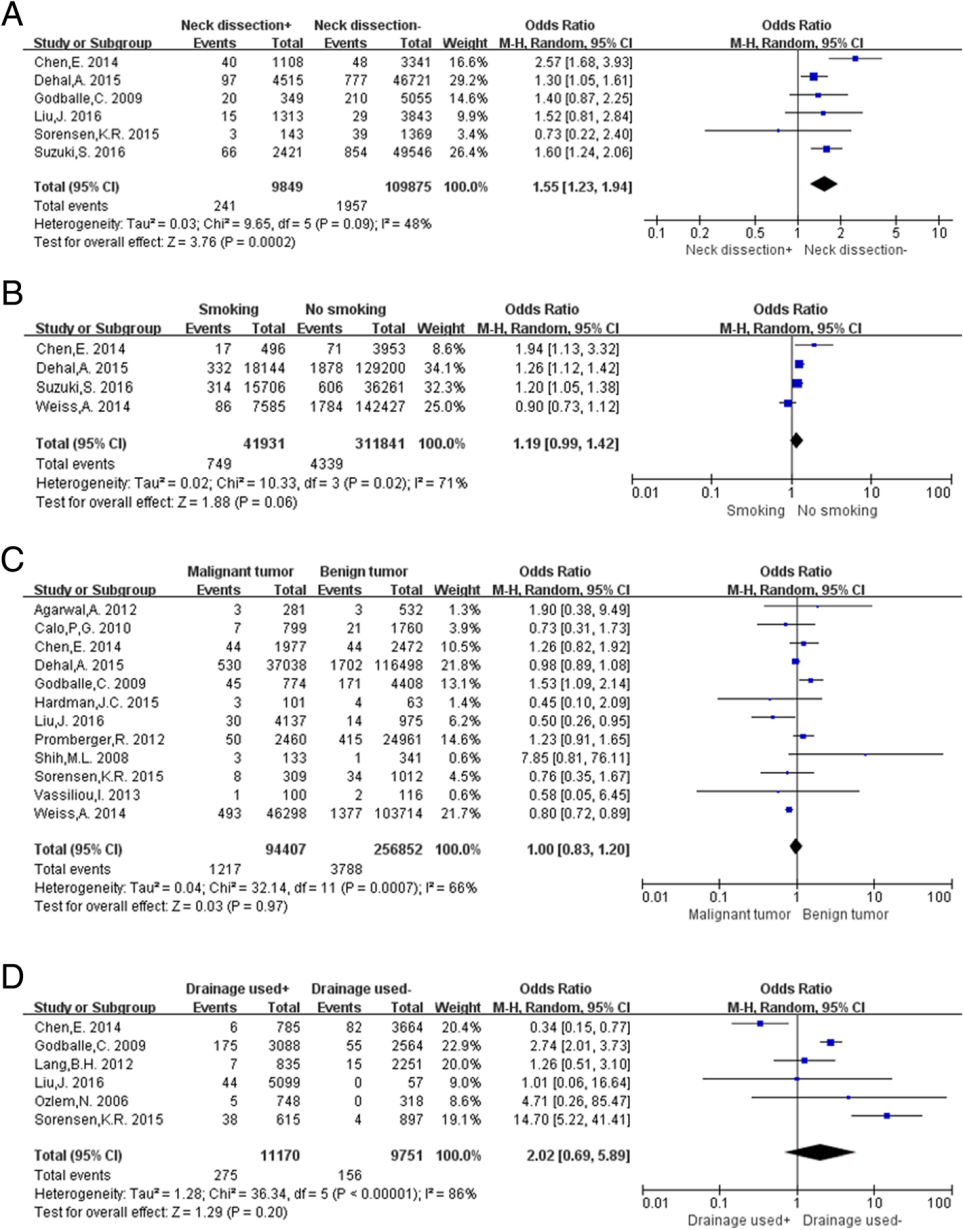
Meta-analysis results of post-thyroidectomy hematoma between the 2 groups. **a** Neck dissection; **b** Smoking status; **c** Tumor character; **d** Drainage used

Data analysis of neck dissection using a random-effects model (*P* = 0.09, I^2^ = 48%) showed that postoperative hematoma was more likely to occur in patients who underwent neck dissection than in those who did not (OR: 1.55, 95% CI: 1.23–1.94, *P* = 0.0002; Fig. 4a).

#### Smoking status

A random-effects model was used for this analysis because of the high heterogeneity in the collected data (*P* = 0.02, I^2^ = 71%). The results showed that there was no significant increase in the incidence of post-thyroidectomy hematoma in patients who smoked (OR: 1.19, 95% CI: 0.99–1.42, *P* = 0.06; Fig. 4b).

#### Tumor characteristics

Twelve studies [6–10, 12, 13, 15, 18–21] were included for this analysis by using a random-effects model t test (*P* = 0.0007, I^2^ = 66%). The tumor was classified as malignant or benign, and we found that the tumor characteristics were not associated with the risk of postoperative hematoma (OR: 1.00, 95% CI: 0.83–1.20, *P* = 0.97; Fig. 4c).

#### Drain placement

Data concerning drain placement were extracted from 6 studies and a random-effects model was used to analyze the data (*P* < 0.00001, I^2^ = 86%). The results showed that the placement of drain devices was not associated with the occurrence of post-thyroidectomy hematoma (OR: 2.02, 95% CI: 0.69–5.89, *P* = 0.20; Fig. 4d).

Pooled outcomes of all factors are shown in Table 2.

**Table 2:** Pooled outcomes of all factors

Factors	No. of studies	Statistical model	MD/OR	95%CI	P vaule	Heterogeneity
Male	12	random-effects	1.86	1.60–2.17	< 0.00001	*P* = 0.03, I^2^ = 48%
Age	6	fixed-effects	4.92	4.28–5.56	< 0.00001	*P* = 0.74, I^2^ = 0%
Graves Disease	8	fixed-effects	1.81	1.60–2.05	< 0.00001	*P* = 0.21, I^2^ = 28%
Hypertension	4	random-effects	2.27	1.43–3.60	0.0005	*P* < 0.00001, I^2^ = 92%
Antithrombotic Drug used	6	fixed-effects	1.92	1.51–2.44	< 0.00001	*P* = 0.20, I^2^ = 32%
Low-volume hospital	5	random-effects	1.32	1.12–1.57	0.001	*P* = 0.0001, I^2^ = 83%
Previous thyroid surgey	7	random-effects	1.93	1.11–3.37	0.02	*P* = 0.003, I^2^ = 69%
Bilateral thyroidectomy	7	fixed-effects	1.19	1.09–1.30	< 0.0001	*P* = 0.12, I^2^ = 40%
Neck dissection	6	random-effects	1.55	1.23–1.94	0.0002	*P* = 0.09, I^2^ = 48%
Smoking status	4	random-effects	1.19	0.99–1.42	0.06	*P* = 0.02, I^2^ = 71%
Malignant tumor	12	random-effects	1.00	0.83–1.20	0.97	*P* = 0.0007, I^2^ = 66%
Drainage used	6	random-effects	2.02	0.69–5.89	0.20	*P* < 0.00001, I^2^ = 86%

*MD* Mean difference, *OR* Odds ratio, *95% CI* 95% confidence interval

## Discussion

Postoperative hematoma is a challenging complication after thyroidectomy, particularly during the period following out-patient surgery. Cervical hematoma formation may lead to airway obstruction or respiratory distress in some cases, and immediate surgical re-intervention is required to avoid asphyxia, cardiac arrest, or even death. Although post-thyroidectomy hematoma is a rare complication, it may be potentially life-threatening once the symptoms develop. As a large proportion of thyroid surgery procedures are performed on an outpatient basis, the avoidance of postoperative hematoma has become a major concern for clinical surgeons [28]. Therefore, we pooled the results of previous studies to identify potential risk factors associated with neck hematoma requiring surgical intervention after thyroidectomy.

Our meta-analysis included 50,171 males and 185,937 females undergoing thyroidectomy for various types of thyroid diseases. The risk of post-thyroidectomy hematoma was 1.86-fold higher in males than in females (OR: 1.86, 95% CI: 1.60–2.17, *P* < 0.00001). The reason for the different incidence between males and females is unclear, but we infer that there are 3 possible causes. First, blood vessels in males are thicker than in females, and therefore, blood flow in the thyroid glands is more abundant. When hemostasis in the neck area is inadequate or surgical ligation loosens, thick vessels can generate more vascular hemorrhage than thin vessels, which increases the likelihood of neck hematoma in males. In addition, smoking, drinking, and hypertension are more common in males [19, 35], all of which can cause vascular changes such as increased vascular brittleness or decreased coagulation. When neck stimulation, such as in emesis, cough, or abrupt neck activity, occurs, vascular rupture and bleeding are more likely to occur, which leads to the formation of hematoma. Finally, some studies suggest that male gender is one of the risk factors of thyroid cancer and males are hence more prone to develop lymph node metastasis [36, 37]; therefore, thyroid surgery may be more complex and the extent of surgery may be wider, and blood vessel damage and bleeding could be greater. This could be a contributing factor for the formation of hematoma.

Several previous articles have reported that age is a risk factor for post-thyroidectomy neck hematoma [3, 4, 7, 10, 12, 22]. In the present study, the average patient age ranged from 46.5 to 56.1 years; the mean age of the hematoma group was greater than that of the non-hematoma group (MD: 4.92, 95%CI: 4.28–5.56, *P* < 0.00001), which suggests that older patients in this age range had a higher risk of suffering postoperative hematoma. This conclusion is consistent with the previous results. We believe that this outcome can be attributed to the increased vascular brittleness in older patients. As individuals age, the blood vessels may become less elastic, which may likely contribute to angiorhagia and decreased vasoconstriction. Eventually, neck hemorrhage or hematoma may occur. However, as the age divisions in previous studies are not consistent, we could not determine which age bracket was most prone to postoperative neck hematoma.

At present, the indications of thyroid surgery for Graves disease include compressive symptoms, uncontrollable hyperthyroidism, large goiter, coexisting malignant tumors, retrosternal or substernal extension, females planning pregnancy, and Graves ophthalmopathy [38]. The advantage of surgery for Graves disease is that definitive treatment can be provided, and relative symptoms can be rapidly alleviated. However, Graves disease is an autoimmune thyroid condition, and the thyroid glands of patients with Graves disease are rich in vascularity [39]. This type of physiological change may increase the risk of intraoperative hemorrhage and may aggravate the influence of surgical vision, which could enhance the difficulty of the surgery or may even lead to an increased incidence of complications. Previous research has shown that using inorganic iodine for several days prior to surgery can decrease intraoperative blood loss by reducing thyroid hormone release and thyroid vascularity, and should hence be recommended for most patients undergoing thyroidectomy for Graves disease [40]. However, most studies included in our research did not include a detailed description of the use of inorganic iodine before surgery, and did not explain whether this preoperative therapy, if regularly performed, could affect the risk of postoperative hematoma in Graves disease. In the present study, treatment with inorganic iodine had to be ignored due to the lack of detailed information. Therefore, we found that there was an increased rate of hematoma formation following thyroidectomy for Graves disease, as compared to other indications for thyroidectomy (OR: 1.81, 95% CI: 1.60–2.05, *P* < 0.00001).

Some researchers believe that postoperative hematoma formation in most cases was probably due to postoperative hypertension (SP > 150 mmHg) [41]. Hypertension may cause vascular stiffness, which increases the likelihood of postoperative hemorrhage; in particular, elevated blood pressure after surgery could increase the risk of hematoma formation. Hence, close monitoring of blood pressure during the first 24 h after surgery and prompt treatment of all manifestations of hypertension with appropriate drugs are recommended [42]. In the present study, we observed an increased risk of hematoma in patients with hypertension (OR: 2.27, 95% CI: 1.43–3.60, *P* = 0.0005). Therefore, care should be taken during the postoperative period in patients with hypertension, particularly in patients in whom antihypertensive therapy has been discontinued preoperatively due to anesthesia. Smooth extubation without significant retching or coughing, as well as the control of both postoperative vomiting and pain to avoid an increase in venous or arterial pressure, are important considerations for minimizing the risk of post-thyroidectomy hematoma [35, 43, 44].

The preoperative use of antiplatelet and/or anticoagulant medications may be another potential risk factor for post-thyroidectomy hematoma. The indications and use of these medications are expanding in older populations [45], as these drugs have been proven to prevent clot formation or platelet aggregation, both of which can affect postoperative hemostasis. Therefore, some surgeons maintain that avoiding antithrombotic drug use 1 week before surgery may decrease the risk of postoperative hemorrhage; these antithrombotic drugs include anticoagulant agents (warfarin, edoxaban, apixaban, rivaroxaban, and dabigatran) and antiplatelet agents (aspirin, clopidogrel, ticlopidine, and cilostazol) [46]. Nevertheless, it is unclear whether the use of these antithrombotic drugs should be suspended and whether a 1-week period is sufficient. In the present analysis, we included all patients who used antithrombotic drugs, regardless of the drug type, dosage, route of medication, and drug outage time. The results of statistical analysis indicated that patients receiving antithrombotic medications had an increased risk of hematoma formation, compared with those not receiving these medications (OR: 1.92, 95% CI: 1.51–2.44, *P* < 0.00001). Due to our limited dataset, we were unable to perform subgroup analysis about the drug type, dosage, route of medication, and drug outage time. Hence, further studies are needed to clarify the impact of these drugs on the risk of postoperative hematoma formation.

Moreover, our research analyzed the relationship between the volume of hospitals and the occurrence of postoperative hematoma. We divided the hospitals into high-volume and low-volume hospitals. High-volume hospitals were defined as centers that performed ≥75 thyroidectomies per year on average, and low-volume hospitals were defined as centers that performed as < 75 thyroidectomies per year. We found that more cases of post-thyroidectomy hematoma occurred in low-volume hospitals (OR: 1.32, 95% CI: 1.12–1.57, *P* = 0.001). The most common explanation for this relationship could be that both surgeon and hospital experience is related to improved patient care—i.e., “practice makes perfect”. Some investigators have noted an independent effect of hospital volume [47], whereas other researchers found that most of the volume–outcome relation can be explained by surgeon experience [48, 49]. Surgeons in high-volume hospitals may have more experience with thyroid surgery and may be better at blood vessel ligature or intraoperative hemostasis, which may explain the low incidence of hematoma in high-volume hospitals.

Re-operative thyroid surgery can be challenging for surgeons. During the primary thyroid surgery, removal of part or the entire gland could distort the anatomy and cause postoperative tissue changes through scarring and fibrosis [50]. Radioactive iodine treatment can make tissues stiffer, woodier, and more likely to bleed [34]. The second or third thyroidectomy in these cases will involve dissection through previously disturbed tissues, which could contribute to a higher hematoma rate. Our study showed that previous thyroid surgery was a risk factor for post-thyroidectomy hematoma (OR: 1.93, 95% CI: 1.11–3.37, *P* = 0.02) [6–8, 16, 17, 20, 24]. Thyroidectomy in patients who have undergone a previous thyroid operation may therefore be safely considered by experienced surgeons.

Hematoma is more likely to occur in patients undergoing a more extensive surgical resection. Compared with unilateral thyroidectomy, bilateral thyroidectomy results in a larger wound and greater tissue injury. Larger wounds and more severe tissue injury may greatly increase the possibility of postoperative hematoma (OR: 1.19, 95% CI: 1.09–1.30, *P* < 0.0001). This explanation may also be responsible for the higher incidence of postoperative hematoma in patients with neck dissection (OR: 1.55, 95% CI: 1.23–1.94, *P* = 0.0002). Compared with simple thyroidectomy, neck dissection involves an additional procedure that requires resection over a larger anatomical area and may be associated with larger injury to the cervical muscles and surrounding blood vessels. A large dead space is often formed following neck dissection and facilitates hematoma formation [12]. Cervical muscles are a common postoperative bleeding site, and hence, they need to be stretched to obtain an essential amount of operating space and surgical vision during thyroidectomy, particularly when deep lymph node dissection is performed. This may lead to injury to small blood vessels on the surface of the muscle, and may increase the risk of postoperative hematoma formation. To avoid overlooking imperceptible bleeding points, a meticulous surgical technique with careful hemostasis is necessary.

It is unclear whether smoking status is a risk factor for post-thyroidectomy hematoma. Some researchers suggested that smokers had an increased postoperative bleeding tendency [19, 35], but others argued that smoking status was not an independent risk factor. Weiss et al. [2, 9, 10, 12] found no association between smoking status and postoperative hematoma, consistent with our conclusion (OR: 1.19, 95% CI: 0.99–1.42, *P* = 0.06). In the smokers in the present study, none of the included studies provided a relative description of the number of cigarettes smoked per day. We ignored the amount of cigarettes smoked, and defined smoking status as smoker (current or former smoker) and never smoker.

Our univariate analysis showed no significant correlation between the smoking status and postoperative hematoma, although the risk of neck hematoma was increased in patients with chronic obstructive pulmonary disease (COPD), diabetes, chronic renal disease, and other underlying diseases with a comorbidity score of ≥3 [9]. Due to the limited data, we were unable to further analyze the comorbidities; however, it is important to control the comorbidities before the operation.

To analyze the relationship between the nature of thyroid tumors and postoperative hematoma, we divided the tumors into benign and malignant, and found that there was no significant difference between them in terms of postoperative hematoma formation (OR: 1.00, 95% CI: 0.83–1.20, *P* = 0.97), inconsistent with previous studies [2, 6]. In several included studies, some researchers classified benign thyroid lesions as benign tumors, which could have affected the outcome. Moreover, in some patients, the final pathological diagnosis may differ from the intraoperative frozen section diagnosis, which could have affected the result.

Drain device placement after thyroidectomy is a routine procedure to drain possible postoperative hemorrhage, which could lead to cervical hematoma formation or even airway compression [51]. Our analysis indicated that routine placement of drain devices did not significantly increase the risk of postoperative hematoma (OR: 2.02, 95% CI: 0.69–5.89, *P* = 0.20). However, previous studies showed that this prevention measure did not have significant postoperative advantages because it could have increased the incidence of infective complications and led to a longer hospital stay [52–54]. Based on these findings, we concluded that although routine drain device placement is a not a risk factor for postoperative hematoma formation, meticulous intra-operative hemostasis should never be ignored.

Besides the above-mentioned analysis, diabetes, alcohol abuse, high body mass index, obesity, multiple nodules, thyroid weight, substernal goiter, history of iodine therapy, anesthesia methods, and new types of hemostatic equipment may be related to postoperative hematoma. However, due to the insufficiency of the literature, we were unable to include all these factors into our meta-analysis. To clarify these factors, additional relative research will be required.

### Limitations

Our meta-analysis has certain limitations. First, in some studies, the definition of hematoma was not clear. Therefore, we clearly defined hematoma as patients who require surgical re-intervention, including return to the operating room or need for bedside monitoring with incision of the skin and evacuation of hematoma. Consequently, studies that did not meet this inclusion criteria were excluded, which means that our analysis may have underestimated the overall incidence of hematoma. Second, only 4 articles meeting the inclusion criteria analyzed the correlation of hypertension and smoking status with postoperative hematoma formation, and hence, the resulting conclusion may not be sufficiently convincing. Third, in the 4 articles that analyzed the volume of hospitals, the definition of hospital volume was inconsistent, and we had to classify hospitals as high-volume and low-volume, which could affect the inconsistency between the conclusion and the objective facts. Fourth, we did not perform a specific classification of thyroidectomy (total thyroidectomy, subtotal thyroidectomy, thyroid lobectomy) and failed to find an association between surgical procedures and postoperative hematoma. Fifth, the studies included in our meta-analysis did not describe whether hemostatic devices such as the Ligasure vessel sealing system and ultrasonic scalpels were used, and therefore, we could not judge whether the new type of hemostatic equipment(e.g. ultrasonic scapel) would reduce the incidence of hematoma. Finally, most of the included studies originated from European and American regions, which might lead to selective bias.

## Conclusions

Our study identified several risk factors for neck hematoma requiring surgical re-intervention after thyroidectomy, including male gender, age, Graves disease, hypertension, antithrombotic agent use, thyroid procedures in low-volume hospitals, previous thyroid operation, bilateral thyroidectomy, and neck dissection. To minimize the occurrence of postoperative hematoma, extensive postoperative monitoring should be performed in patients with multiple identifiable risk factors, particularly when thyroidectomy is performed on an outpatient basis.

## Data Availability

All data generated or analyzed during this study are available from all the included studies from PubMed and CNKI databases.

## Abbreviations

CI: Confidence interval
CNKI: China national knowledge infrastructure
COPD: Chronic obstructive pulmonary disease
MD: Mean difference
NOS: Newcastle Ottawa Scale
OR: Odds ratio
PRISMA: Preferred Reporting Items for Systematic Review and Meta-Analyses
